# Unilateral Left-Sided High-Altitude Pulmonary Edema Following Rapid Ascent to a City in the Himalayas

**DOI:** 10.7759/cureus.69068

**Published:** 2024-09-10

**Authors:** Ananthu Sobhanan, Rahul Tyagi, Jitendra K Tiwari, Nidhisha Kanakambujan

**Affiliations:** 1 Emergency Department, Army Institute of Cardiothoracic Sciences, Pune, IND; 2 Pulmonary Critical Care and Sleep Medicine Department, Army Institute of Cardiothoracic Sciences, Pune, IND; 3 Radiology, General Hospital, Leh, IND; 4 Internal Medicine, Manchester University NHS Trust, Manchester, GBR

**Keywords:** acute breathlessness, altitude, high altitude pulmonary edema, lung opacities in xray, unilateral pulmonary edema

## Abstract

High-altitude pulmonary edema (HAPE) is a medical emergency commonly observed in people ascending to altitudes above 9000 ft. The condition predominantly presents with bilateral clinical and radiological findings. In rare cases, where unilateral lung involvement is seen, a right lung predilection is usually noted. We present a case of HAPE with unilateral left lung involvement in a young man upon reintroduction to a high-altitude area. He presented with dyspnea at rest and was found to have severe hypoxemia and tachycardia. Clinical and radiological evidence showed unilateral left lung findings. After excluding other plausible causes, it was concluded to be a case of HAPE. Following treatment, his symptoms resolved, and he was discharged on the fifth day after admission. This case highlights the necessity of awareness related to the uncommon presentations of HAPE since this condition can be completely reversed with a good patient outcome if suspected and treated early.

## Introduction

High-altitude pulmonary edema (HAPE) is a life-threatening condition observed upon sudden ascent to high-altitude regions (> 9000 feet) [1,2]. With the increasing trends in tourism, pilgrimage, and military-related activities in high-altitude areas, the incidence of HAPE is on the rise [3]. This emphasizes the need for research on high-altitude disorders, as current research data are limited. Typically, HAPE manifests with the involvement of both lungs, which is evident from bilateral clinical and radiographic findings [2]. However, in some cases, it may present with unilateral findings, making it crucial to differentiate HAPE from infective and thrombotic causes [4]. Here, we present a case of atypical HAPE observed in a young man upon induction to a high-altitude area.

## Case presentation

A young male in his late twenties, with no known previous co-morbidities, was rapidly inducted to a high-altitude region (11,500 ft above sea level) via air. He hailed from southern coastal India and was fit and well before he started his journey. He did not report any history of alcohol consumption or smoking.

On the second day following his induction, he presented to the emergency room of the acclimatization center with a sudden onset of dyspnea at rest. He also complained of a throbbing headache, moderate in intensity, localized bilaterally in the occipital region. Additionally, he reported a history of nonproductive cough, fatigue, reduced appetite, and difficulty sleeping, all of one day's duration. He did not report any history of fever, vomiting, muscle pain, or focal neurological symptoms.

On clinical evaluation, his blood pressure was recorded as 122/78 mmHg in the right arm in a sitting posture. He was afebrile (body temperature 36.6 degrees Celsius ), with mild tachycardia (pulse rate of 100 beats per minute) and tachypnea (respiratory rate of 32 cycles per minute). His arterial oxyhemoglobin saturation was 66% on breathing ambient air. Examination of his respiratory system revealed diffuse inspiratory crackles heard in the left infrascapular and axillary areas. The cardiovascular examination was normal. Examination of the extremities did not reveal evidence of erythema, edema, or tenderness.

**Figure 1:**
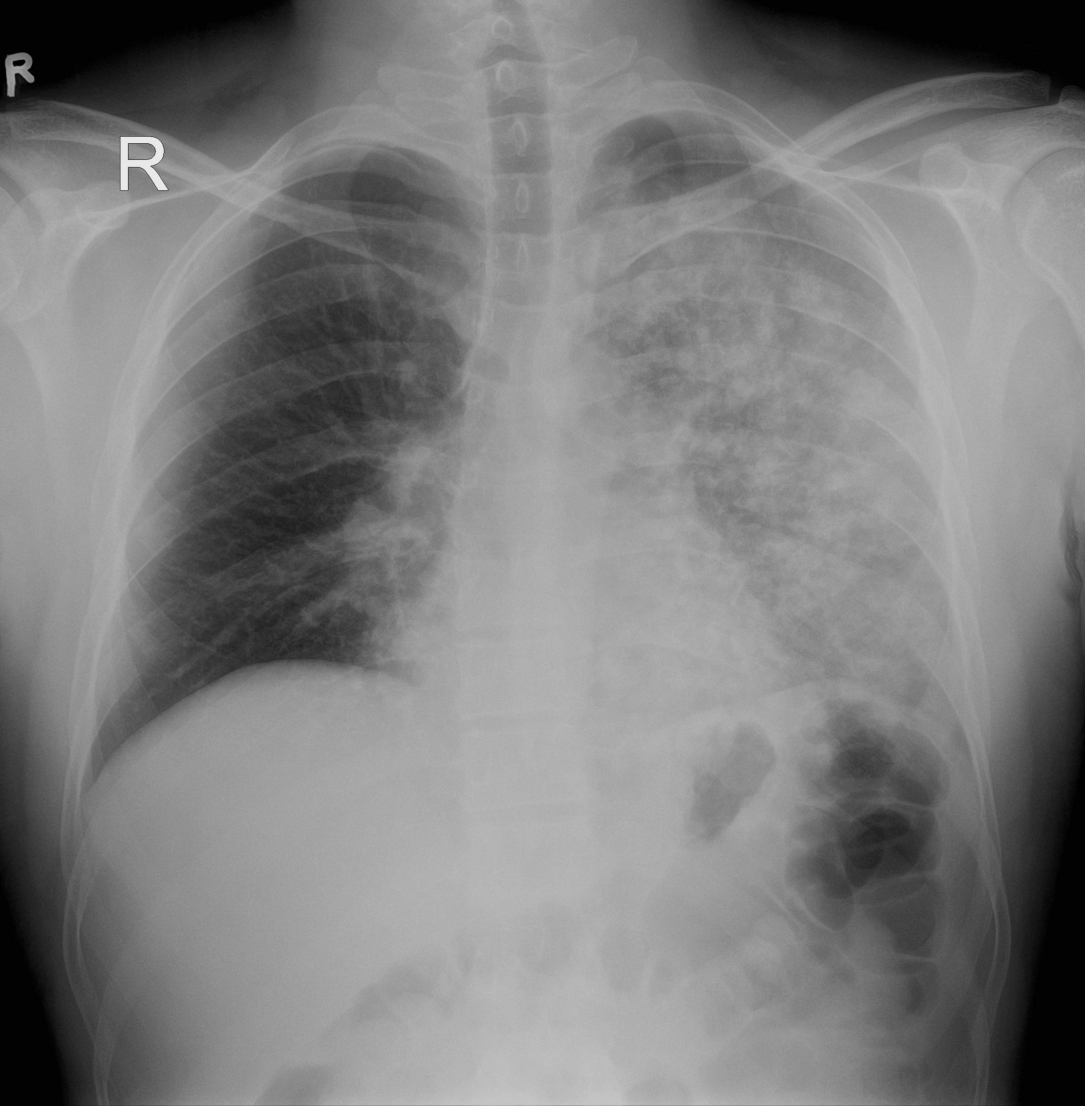
Posteroanterior (PA) chest radiograph on admission showing unilateral left-sided nonhomogenous alveolar opacities with patchy areas of air space consolidations

His complete blood count, serum electrolytes, urea, creatinine, total bilirubin, aspartate aminotransferase (AST)/alkaline phosphatase (ALP), and uric acid were within normal limits. Blood samples were also negative for fibrin degradation products, and the D-dimer value was 433 ng/ml. Blood was taken for culture and sensitivity, which later came back negative. His rapid antigen test for COVID-19 was negative. ECG tracing showed sinus tachycardia. The echocardiogram revealed a normal ejection fraction (EF) of 60-65% with no structural abnormalities in the heart. A posteroanterior (PA) view chest radiograph showed nonhomogeneous, alveolar opacities with patchy areas of airspace consolidations in the lower, middle, and upper zones of the left lung (Figure 1).

He was immediately started on supplemental oxygen at a rate of 6 liters/min via a face mask. Additionally, he received injections of dexamethasone 6 mg IV, Tab nifedipine 20 mg PO, and Tab acetazolamide 250 mg PO twice daily. Deinduction to a lower altitude was not possible due to extreme weather conditions. Therefore, he was admitted to the nearest secondary care hospital and treated at the same altitude (11,500 ft).

**Figure 2:**
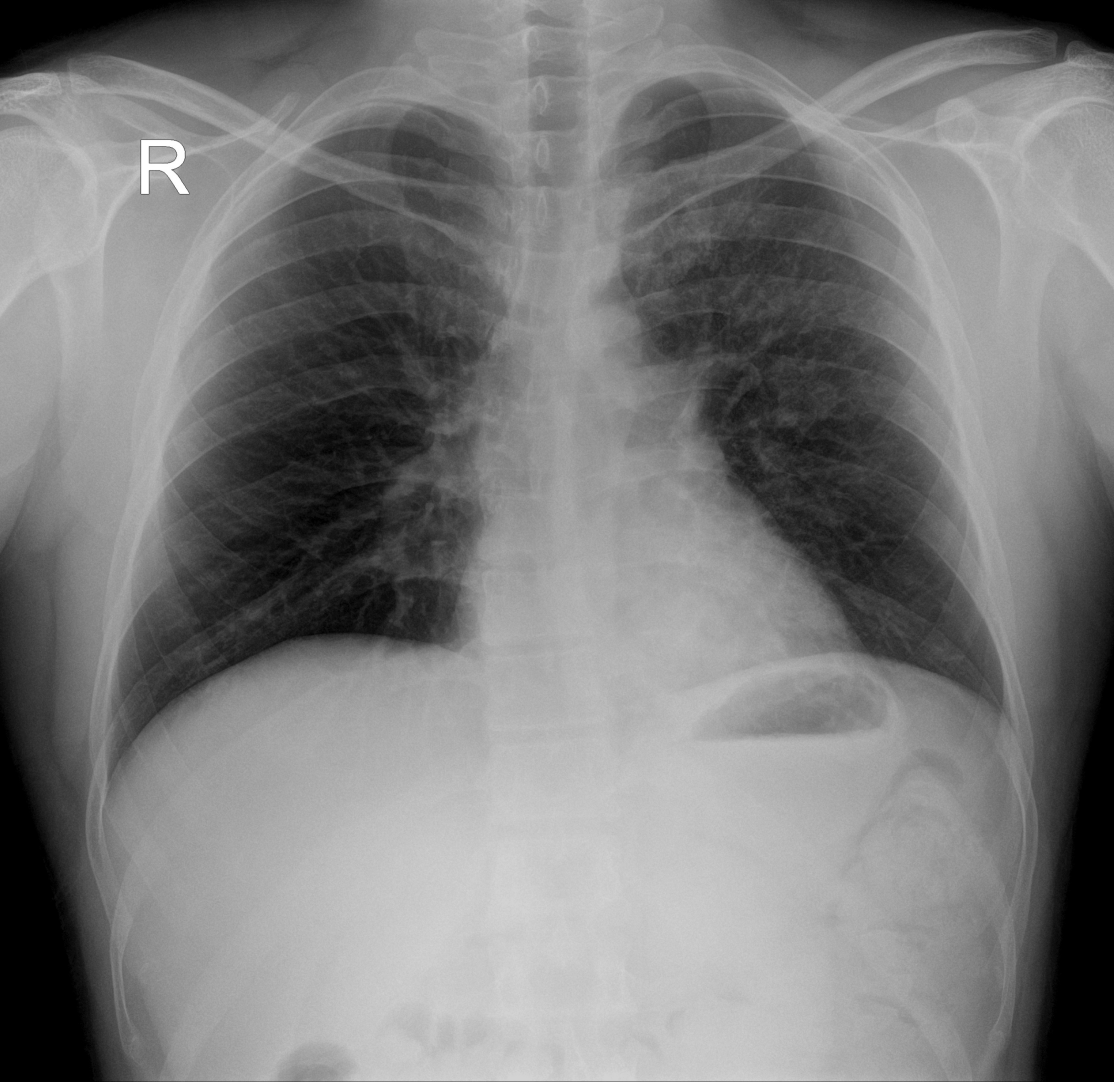
Posteroanterior (PA) chest radiograph on the third day after admission showing complete resolution of opacities

The patient showed gradual progressive improvement over the next few days and did not warrant further referral to a higher center. His oxygen requirement reduced, and he was weaned off supplemental oxygen over the next three days. His dyspnea, headache, and cough also resolved, with improvement in sleep and appetite. A repeat posteroanterior (PA) chest radiograph on the third day after admission showed complete resolution of opacities (Figure 2). He was discharged on the fifth day and was advised to avoid ascent to higher altitudes in the next six weeks.

## Discussion

High-altitude pulmonary edema is a condition that affects healthy individuals when they ascend to the hypobaric hypoxic conditions of high-altitude regions. It is a potentially fatal form of non-cardiogenic pulmonary edema with up to a 50% mortality rate in untreated conditions [1,2,5]. Data and case studies on high-altitude-induced pulmonary edema are scarce. Interestingly, due to military activities and adventure-related tourism, human interference in these previously unoccupied unphysiological terrains is rising.

The exact pathophysiology of HAPE is still not clear. The most favored theory, according to the literature, is hypoxia-induced diffuse patchy vasoconstriction and sympathetic activation. Precipitating factors contributing to HAPE include a sudden and severe change in barometric pressure along with decreased partial pressure of oxygen. These factors cause uneven vasoconstriction, resulting in increased capillary pressure and capillary permeability, causing fluid collection in the lungs. Other theories include inflammation in the lungs and a genetic propensity to develop HAPE [5].

HAPE is a clinical diagnosis and can develop within hours to days of ascent to high altitude, usually occurring within the first week. Though patients initially present with symptoms such as weakness, breathlessness on exertion, and cough, it may rapidly progress to dyspnea at rest, orthopnea, productive cough, and chest pain. Symptoms of acute mountain sickness, such as headache, gastrointestinal symptoms, reduced appetite, and sleep disturbances, can also coexist with HAPE. Clinical features include crackles on auscultation, cyanosis, hypoxemia, and pink-frothy sputum [5].

In this case study, the presumed differential diagnosis included pneumonia, aspiration pneumonia, pulmonary embolism, pulmonary hemorrhage, and unilateral pulmonary edema. The absence of fever and productive cough, coupled with a normal leukocyte count despite the presence of opacities in his left lung, made it unlikely to be pneumonia. Normal fibrin degradation products (FDP) and D-dimer reduced the probability of the diagnosis of pulmonary embolism. Since he had no history of syncopal episodes, altered sensorium, seizures, vomiting, or any neurological complaints causing aspiration, the likelihood of aspiration pneumonia was low. Lack of predisposing conditions and no complaints of hemoptysis minimized the chances of alveolar hemorrhage. Lastly, the absence of any signs pertaining to cardiovascular disorders, as well as a normal ECG and echocardiography decreased the chances of any apparent cardiac disease. The unavailability of chest computed tomography facilities in this remote area limited further investigations to explore the causes convincingly and definitively. After rationally excluding other possibilities with the available data and factoring in his recent travel history, we considered the diagnosis of pulmonary edema precipitated by rapid ascent to a high-altitude area.

Current data shows that bilateral chest findings are predominantly observed in HAPE [6-8]. Even on rare occasions where unilateral pulmonary edema has been observed, right lung predilection and predominance were noted more than left [4,9,10]. A possible explanation is the poorer lymphatic drainage of the right lung by the small-caliber, right bronchomediastinal trunk in comparison with that of the left lung by the large-caliber thoracic duct [11]. However, this case contradicts both the above observations. Unilateral left-sided chest findings were auscultated on examination, which correlated with the unilateral left lung opacities seen in the chest radiograph. Disease progression and resolution of symptoms by following HAPE protocols were consistent with the provisional diagnosis, consequently making this an atypical presentation of HAPE.

Literature describing unilateral left-sided high-altitude pulmonary edema is exceedingly scarce. The pathophysiology for atypical findings of HAPE remains unknown. Plausible mechanisms, such as asymmetrical perfusion due to unilateral pulmonary artery hypoplasia/agenesis or unilateral embolism, and cardiac disorders like mitral regurgitation, have been suggested [11-14]. However, these were deemed unlikely based on normal investigation findings and the absence of signs or symptoms during examination. Previous trauma, damaged lung tissue, or prolonged lateral decubitus positioning could possibly explain the left lung findings [8].

## Conclusions

The incidence of HAPE is increasing, propelled by the growth in tourism and military-related activities. In the remote settings of high-altitude areas where HAPE commonly occurs, timely and accurate diagnosis is crucial. Atypical presentations of high-altitude pulmonary edema can present challenges for early diagnosis. In areas with limited medical resources, radiographs and clinical findings may serve as the main tools for diagnosis. Therefore, it is imperative for physicians to be familiar with both typical and atypical presentations of HAPE, as early diagnosis and intervention are paramount in HAPE cases.
